# Calcium Signaling and Contractility in Cardiac Myocyte of Wolframin Deficient Rats

**DOI:** 10.3389/fphys.2019.00172

**Published:** 2019-03-13

**Authors:** Michal Cagalinec, Alexandra Zahradníková, Alexandra Zahradníková, Dominika Kováčová, Ludovit Paulis, Simona Kureková, Matej Hot’ka, Jana Pavelková, Mario Plaas, Marta Novotová, Ivan Zahradník

**Affiliations:** ^1^Department of Cellular Cardiology, Institute of Experimental Endocrinology, Biomedical Research Center, University Science Park for Biomedicine, Slovak Academy of Sciences, Bratislava, Slovakia; ^2^Institute of Molecular Physiology and Genetics, Centre of Biosciences, Slovak Academy of Sciences, Bratislava, Slovakia; ^3^Institute of Biomedicine and Translational Medicine, Faculty of Medicine, University of Tartu, Tartu, Estonia; ^4^Institute of Pathophysiology, Faculty of Medicine, Comenius University, Bratislava, Slovakia; ^5^Institute of Normal and Pathological Physiology, Centre of Experimental Medicine, Slovak Academy of Sciences, Bratislava, Slovakia; ^6^Department of Neurophysiology and Neuropharmacology, Center of Physiology and Pharmacology, Medical University of Vienna, Vienna, Austria

**Keywords:** calcium current, calcium transient, contractility, cardiac myocyte, Wolfram syndrome, wolframin

## Abstract

Wolframin (Wfs1) is a membrane protein of the sarco/endoplasmic reticulum. Wfs1 mutations are responsible for the Wolfram syndrome, characterized by diabetic and neurological symptoms. Although Wfs1 is expressed in cardiac muscle, its role in this tissue is not clear. We have characterized the effect of invalidation of Wfs1 on calcium signaling-related processes in isolated ventricular myocytes of exon5-Wfs1 deficient rats (Wfs1^-e5/-e5^) before the onset of overt disease. Calcium transients and contraction were measured in field-stimulated isolated myocytes using confocal microscopy with calcium indicator fluo-3 AM and sarcomere length detection. Calcium currents and their calcium release-dependent inactivation were characterized in whole-cell patch-clamp experiments. At 4 months, Wfs1^-e5/-e5^ animals were euglycemic, and echocardiographic examination revealed fully compensated cardiac function. In field-stimulated isolated ventricular myocytes, both the amplitude and the duration of contraction of Wfs1^-e5/-e5^ animals were elevated relative to control Wfs1^+/+^ littermates. Increased contractility of myocytes resulted largely from prolonged cytosolic calcium transients. Neither the amplitude of calcium currents nor their voltage dependence of activation differed between the two groups. Calcium currents in Wfs1^-e5/-e5^ myocytes showed a larger extent of inactivation by short voltage prepulses applied to selectively induce calcium release-dependent inactivation of calcium current. Neither the calcium content of the sarcoplasmic reticulum, measured by application of 20 mmol/l caffeine, nor the expression of SERCA2, determined from Western blots, differed significantly in myocytes of Wfs1^-e5/-e5^ animals compared to control ones. These experiments point to increased duration of calcium release in ventricular myocytes of Wfs1^-e5/-e5^ animals. We speculate that the lack of functional wolframin might cause changes leading to upregulation of RyR2 channels resulting in prolongation of channel openings and/or a delay in termination of calcium release.

## Introduction

Wolfram syndrome (WS; OMIM 222300) is a rare hereditary disorder, first identified as a clinical entity separate from the juvenile type of diabetes mellitus by Wolfram and Wagener (1938). WS is characterized by symptoms including diabetes insipidus (DI), diabetes mellitus (DM), optical atrophy (OA), and deafness (D), and therefore it is termed also DIDMOAD (Barrett et al., 1995). Most WS cases are caused by recessive mutations of the *Wfs1* gene, located on chromosome 4p16.1. The Wfs1 protein is highly expressed in the brain, heart and pancreatic β-cells (Inoue et al., 1998; Yamada et al., 2006); pancreas and brain represent the crucial organs responsible for most of clinical symptoms in WS. Cardiac symptoms of WS were not originally recognized; however, emerging clinical findings include heart malformations as well as sinus tachycardia, atrial or ventricular arrhythmias (Medlej et al., 2004; Fabbri et al., 2005; Ganie et al., 2011; Korkmaz et al., 2016). The high expression of Wfs1 in the heart tissue, and the cardiac symptoms identified until now suggest functional importance of Wfs1 in the heart.

Although the *Wfs1* gene was identified 20 years ago (Inoue et al., 1998), the function of Wfs1 has not been resolved yet, and little is known about its 3D-structure. Wfs1 is composed of 890 amino acids (MW of ≈100 kDa), and was shown to reside on the membrane of endoplasmic reticulum (ER; Takeda et al., 2001). It was proposed to contain at least nine transmembrane helices (Hofmann et al., 2003), and the amino- and carboxy- terminals were shown to be located in the cytoplasm and in the lumen of ER, respectively. Wfs1 seems to exist predominantly as a tetramer (Hofmann et al., 2003), and ion channel activity was observed after reconstitution of microsomes of Wfs1-expressing Xenopus oocytes in lipid bilayers (Osman et al., 2003). However, homology modeling studies (Qian et al., 2015; Safarpour Lima et al., 2016) produced structures that lack clear channel-forming helices.

Wfs1 was suggested to participate in the response of cells to ER stress: In Wfs1-transfected COS7 cells, Wfs1 negatively regulated the activating transcription factor 6α (ATF6α), a key transcription factor involved in ER stress signaling, and stabilized the E3 ubiquitin ligase HRD1 (Fonseca et al., 2010). In line with this, Cagalinec et al. (2016) have shown that overexpression of Wfs1 leads to massive activation of the key factors of ER stress, namely, ATF6, ATF4, and XBP1 in primary cultured rat cortical neurons. Silencing of Wfs1 by specific shRNA in neurons also induced increased expression of these factors but only to a moderate extent. ER stress caused by Wfs1 deficiency was implicated also in the disruption of β-cell function (Morikawa et al., 2017; Ohta et al., 2017). Wfs1 was also suggested to participate in calcium handling: silencing of Wfs1 by specific shRNA in neurons resulted in depression of calcium transients and of Ca^2+^ release from the ER (Cagalinec et al., 2016). Moreover, expression of Wfs1 in HEK293 cells has been shown to positively modulate Ca^2+^ levels in the ER by increasing the rate of Ca^2+^ uptake (Takei et al., 2006). In addition, Wfs1 co-immunoprecipitates with SERCA, the pump transporting Ca^2+^ from cytosol to lumen of the reticular membrane system (Zatyka et al., 2015). Depletion of Wfs1 led to decreased and delayed cytosolic Ca^2+^ elevations in response to glucose stimuli (Ishihara et al., 2004) and to increased expression of SERCA in β-cells and β-cell lines (Zatyka et al., 2015). Wfs1 has been shown as a molecular partner of calmodulin (Yurimoto et al., 2009) and affected the function of the calcium-dependent protease calpain2 (Lu et al., 2014). In addition, it has been demonstrated recently that Wfs1 forms a complex with neuronal calcium sensor 1 (NCS1) and inositol 1,4,5-trisphosphate receptor (IP3R) to promote Ca^2+^ transfer between the ER and mitochondria in WS patient fibroblasts (Angebault et al., 2018). All these facts demonstrate strong involvement of Wfs1 in calcium signaling and ER-stress mediated pathways.

To understand the role of Wfs1 on the cellular, organ and body level, a Wfs1 deficient mouse model (Luuk et al., 2009) and a Wfs1 loss-of-function rat model (exon5-Wfs1 deficient; Wfs1^-e5/-e5^; Plaas et al., 2017) were developed. In the latter, DM develops at the age of 13 months (Plaas et al., 2017).

Since calcium ions are the sole trigger for myocyte contraction and Wfs1 is strongly involved in calcium metabolism, in this work we evaluated calcium transients and contractility of left ventricular myocytes freshly isolated from the exon5-Wfs1 deficient (Wfs1^-e5/-e5^) rats. To assess whether the cardiac complications in this model develop independently from insulin deficiency, in this study we have studied animals at the age of 4 months, i.e., before the development of hyperglycemia.

## Materials and Methods

### Experimental Model

The impact of Wfs1 malfunction on calcium signaling in cardiac myocytes was studied using the Wfs1 loss-of-function (exon5 deficiency, Wfs1^-e5/-e5^) rat model (Plaas et al., 2017). We used animals at the age of 4 months to avoid complications due to involvement of diabetes.

Rats were kept in groups of two to three in polypropylene cages under a standard 12:12 h light/dark regime at a temperature of 22 ± 2°C and 60–70% humidity. A standard balanced pellet diet and tap water were provided *ad libitum*. The genotype of rats in the control group (Wfs1^+/+^; *n* = 17) and the experimental group (Wfs1^-e5/-e5^; *n* = 19) was verified by PCR (see below). The number of animals used in individual experiments is given together with the data. All experiments conformed to the European directive 2010/63/EU and to Act No. 377/2012 of the Government of the Slovak Republic, were carried out in compliance with the guidelines for the care and use of laboratory animals, and were approved by the Ethical committee of the Centre of Biosciences, Slovak Academy of Sciences and by the State Veterinary and Food Administration of the Slovak Republic (approval No. Ro-1007/16-221 and Ro-2498/18-221a). Rats were sacrificed under full anesthesia by exsanguination. Before chest opening, rats were heparinized (5000 U/kg i.p.) and deeply anesthetized with sodium pentobarbital (100 mg/kg i.p.) until cessation of the paw withdrawal reflex and corneal reflex. Excised hearts attached to the cannula were mounted on the Langendorff set-up for isolation of myocytes, or for whole heart fixation.

The genotype of rats was verified using primers from Plaas et al. (2017), namely, rwfs_zf_genoR1 (5′-AAGAGTGGGTATGGTGCTGG-3′) and rwfs_zf_genoF1 (5′-AGAAGTGGCTACCCAGGGAT-3′). The PCR product was separated in 1.5% agarose gel. A single, 333 (*Wfs1^+/+^*) or 149 bp (*Wfs1*^-e5/-e5^) long band was detected in every animal.

### Echocardiography

Transthoracic echocardiography (Liu and Rigel, 2009) was carried out before cardiotomy and isolation of cardiac myocytes. The examination was performed using a GE Medical Vivid E9 (GE Healthcare, Horten, Norway) using a 10-MHz matrix probe (ML6-15). The rat under sodium pentobarbital anesthesia was placed in supine position on a warming pad (37°C) and the anterior chest was shaved. Left ventricular function and structure were assessed from parasternal short axis views and from the four-chamber apical view. LV end-systolic and end-diastolic diameters and posterior wall thickness during systole and diastole were measured from two-dimensional anatomical M-mode in parasternal short axis view by the leading edge method. LV ejection fraction was calculated by using the Teichholz formula. Aortic outflow was acquired using pulsed wave Doppler. Left ventricular diastolic function was assessed using transmitral flow parameters—the ratio of peak velocity of early (E) and late (A) diastolic filling—from the four-chamber apical view with conventional pulsed wave Doppler. Each measurement was obtained from three consecutive cardiac cycles.

### Blood Glucose Measurements

Blood samples were taken from the tail vein on the day of the experiment after induction of anesthesia and before echocardiographic examination. Glycemia was measured by the glucose oxidase method (Beckman, United States) as described previously (Kurdiova et al., 2014).

### Solutions

*Ripa buffer* (in mmol/l): 150 NaCl, 25 Trizma base (pH 7.6), supplemented with 1% deoxycholate, 0.1% SDS, 1% Triton X-100, and 50 μl/ml protease inhibitor cocktail (Roche Diagnostics, Indianapolis).

*PBST solution* (in mmol/l): 150 NaCl, 3 KCl, 2 KH_2_PO_4_, 8 Na_2_HPO_4_, supplemented with 0.1% Tween-20.

*1Ca Tyrode solution* (in mmol/l): 135 NaCl, 5.4 KCl, 5 MgCl_2_, 1 CaCl_2_, 0.33 NaH_2_PO_4_, 10 HEPES; pH adjusted to 7.25 with 1 mol/l NaOH, osmolarity 300 mosm/l.

*0Ca Tyrode solution* (in mmol/l): 135 NaCl, 5.4 KCl, 5 MgCl_2_, 0.003 CaCl_2_, 0.33 NaH_2_PO_4_, 10.0 HEPES; pH adjusted to 7.25 with 1 mol/l NaOH, osmolarity 300 mosm/l.

*Supplemented 0Ca Tyrode solution* was prepared from 0Ca Tyrode solution by adding (in mmol/l): 10 glucose, 10 creatine, 10 taurine; pH adjusted to 7.25 with 1 mol/l NaOH, osmolarity 330 mosm/l.

*Low-calcium Tyrode solution* was prepared from the Supplemented 0Ca Tyrode solution by adding 50 μmol/l CaCl_2_.

*Enzymatic solution* was prepared from the low-calcium Tyrode solution by adding 0.05–0.2 mg/ml Liberase TM (Roche, Cat. No. 05 401 127 001).

*External bath solution* (in mmol/l): 135 NaCl, 5.4 CsCl, 10 HEPES, 5 MgCl_2_, 0.33 NaH_2_PO_4_, 1 CaCl_2_, 0.01 IBMX, 0.02 TTX.

*Internal solution* (in mmol/l): 135 CsCH_3_SO_3_, 10 CsCl, 10 HEPES, 3 MgSO_4_, 3 ATPNa_2_, 0.05 cAMP, 1 EGTA, and 0.1 Fluo-3.

*Perfusion solution* was prepared from the *Supplemented 0Ca Tyrode* by adding an aliquot of 1 mol/l CaCl_2_ stock solution (Merck) to a final concentration of 1.2 mmol/l CaCl_2_.

*Caffeine solution* was prepared from *Perfusion solution* by adding caffeine at a final concentration of 20 mmol/l.

### Western Blotting

Ventricular cardiomyocytes were lyzed in Ripa buffer. The samples were incubated for 45 min on ice. Total extracts were cleared by centrifugation for 10 min at 4°C at 14,000 × *g* and assayed for protein content by Lowry’s method. Aliquots from each cell lysate containing 25 μg of protein were separated by SDS-PAGE on an 8% gel and transferred to nitrocellulose membrane. Membranes were blocked for 2 h with 5% skim milk in PBST solution, and then incubated with the primary antibody (suspended in 5% skim milk-PBST) overnight or 4 h at room temperature. Primary antibodies used were anti-SERCA2 (1:1000, SC-376235, Santa Cruz) and anti-GAPDH (1:1000, MAB374, Merck-Millipore). Membranes were washed with PBST (three times, 10 min each) and incubated with HRP anti-mouse antibody (1:2000, W4021, Promega) for 1.5 h at room temperature. The bands were visualized with a chemiluminescence immunodetection system (Amersham Biosciences, Piscataway, NJ, United States). Images were analyzed with the Origin software (OriginLab, Ver. 9) by subtracting the background, integrating the signal in each band, and normalizing the SERCA signal to the GAPDH signal in each lane.

### Electron Microscopy

Samples of left ventricular muscle were prepared for morphological analysis as described previously (Mikusová et al., 2009). In brief, the hearts of experimental animals in deep anesthesia were rapidly excised, mounted on a Langendorff setup, and perfused with 1Ca Tyrode solution for 3 min, with 0Ca Tyrode solution for 3 min, and finally with 2% glutaraldehyde in cacodylate buffer (in mmol/l: 150 Na-cacodylate, 2 CaCl_2_, pH 7.3) for 5 min. Three samples of left ventricular muscle per animal (five animals per group) were dissected, exposed to 2% glutaraldehyde in cacodylate buffer for 45 min, postfixed by 1% osmium tetroxide in cacodylate buffer for 30 min, and contrasted with 1% aqueous solution of uranyl acetate. After dehydration in graded ethanol series and propylene oxide, the samples were embedded in Durcupan blocks (ACM Fluka, Sweden). Ultrathin (58–60 nm) longitudinal sections were cut from each block at three distant levels using an ultramicrotome (Power-Tome MT-XL, RMC/Sorvall, Tucson, United States) and mounted on formvar-coated copper grids. The sections were contrasted with lead citrate and examined with a JEM 1200 electron microscope (Jeol, Tokyo, Japan) at 80 kV. The images were recorded with a CCD camera (Gatan DualVision 300W). The endomembrane distribution and ultrastructure were studied at magnification of 2,000–100,000×.

### Isolation of Cardiac Myocytes

Left ventricular cardiac myocytes were isolated from 6 Wfs1^+/+^ and 8 Wfs1^-e5/-e5^ male rats at the age of 4 months. The hearts of experimental animals in deep anesthesia were rapidly excised, cannulated, and mounted on a Langendorff perfusion system. The heart was retrogradely perfused first with 1Ca Tyrode solution until the blood was completely washed out. This was then replaced with Supplemented 0Ca Tyrode solution and perfused for further 5 min. After this, the heart was perfused with Enzymatic solution until the tissue became marble red-white (usually 6–8 min). All solutions were oxygenated and equilibrated at 37°C. During perfusion, the heart was positioned inside a heating chamber maintaining the temperature at 37°C. The heart was then taken off from the perfusion system, the atria and the right ventricle were discarded and the left ventricle was cut gently into several small pieces. The tissue was filtered through a nylon mesh (100 μm). The filtrate containing the dissociated myocytes was gently centrifuged at 50 × *g* for 1 min, the supernatant was discarded and replaced with 1.5 ml low-calcium Tyrode solution. The tissue that remained on the filter was further enzymatically digested for further 5 min at 37°C with Enzyme solution, in which collagenase activity was reduced using BSA (1% w./v.). This procedure was repeated four to five times. The batch with the highest yield of viable myocytes was then processed further by increasing the concentration of CaCl_2_ in low-calcium Tyrode solution to 100, 500, and 1000 μmol/l in three 15-min steps to maintain the myocytes calcium tolerant. All experiments were performed within 8 h after isolation.

### Patch Clamp

Myocytes were whole-cell patch clamped using patch-pipettes of 1.6–1.8 MΩ filled with Internal solution and a VE-2 amplifier (Alembic Instruments, Canada), a Digidata 1320A A/D converter, and pClamp software (Ver. 10, all from Axon Instruments, United States). Cell capacitance and series resistance were compensated to 50–85%. Myocytes were kept at a -50 mV holding potential. The myocytes were kept in a phosphorylated state by the use of ATP and cAMP in the Internal solution and of the membrane-permeable phosphodiesterase inhibitor IBMX in the External solution (Zahradnik and Palade, 1993). Calcium currents elicited by voltage pulses applied by specific protocols were filtered at 10 kHz and sampled at 20 kHz. Current–voltage curves were measured by applying a series of 70-ms test pulses to -40 to +50 mV in 10-mV increments once in 30 s. Inactivation of I_Ca_ was assessed by two types of two-pulse protocols (Zahradníková et al., 2004). *Protocol 1* consisted of a series of twin pulses, applied at 30-s intervals, where the prepulses (duration 5 ms, prepulse potential increasing from -50 to +40 mV by 10 mV increments) were followed by a constant 70-ms test pulse to 0 mV delivered 30 ms after the prepulse. This protocol was used to determine the voltage dependence of the voltage-, current-, and calcium release-dependent components of I_Ca_ inactivation. *Protocol 2* consisted of a series of twin pulses, applied at 30-s intervals, where the prepulses (prepulse potential +60 mV, duration increasing from 0 to 4.5 ms by 0.5 ms) were followed by a constant 70-ms test pulse to 0 mV delivered 30 ms after the prepulse. This protocol served for determination of the calcium release-dependent component of I_Ca_ inactivation. In both inactivation protocols, the fraction of non-inactivated calcium current was calculated for each prepulse as the ratio of the peak amplitude of test I_Ca_ to the peak amplitude of the test I_Ca_ in the absence of the prepulse.

### Confocal Imaging

**FIGURE 1:**
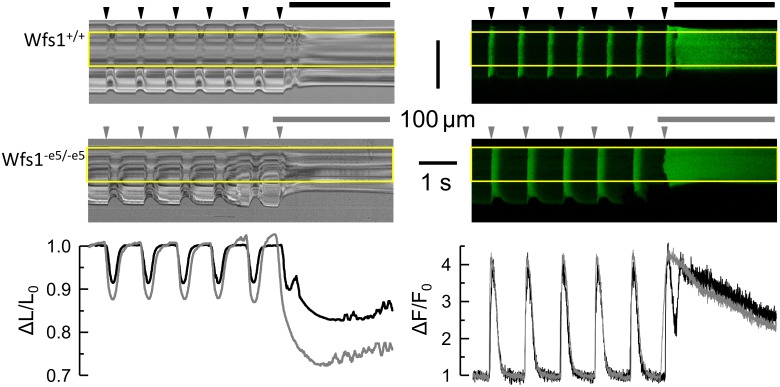
Typical records of sarcomere length and cytosolic Ca^2+^ in response to field stimulation. *Left column* – transmitted light records of line-scans along the myocyte long axis. Deflections correspond to contractions of the myocyte. *Right column* – fluorescence records of calcium signal in confocal line-scans corresponding to the contraction records (left). The yellow rectangles denote the analyzed region. *Top row* – Wfs1^+/+^ myocyte. *Middle row* – Wfs1^-e5/-e5^ myocyte. Arrowheads – the timings of field stimuli. Horizontal lines – applications of caffeine. *Bottom row* – the time course of changes in the relative sarcomere length (left) and cytosolic calcium signals (right) corresponding to the top and middle row records (black – Wfs1^+/+^, gray – Wfs1^-e5/-e5^).

Myocytes were loaded with the calcium indicator fluo-3 AM (1 μmol/l) for 15 min at room temperature. Fluo-3-labeled myocytes suspended in the experimental chamber were left to settle on the coverglass for 1 min and then perfused at a rate of 1 ml/min with Perfusion solution and electrically paced to contract at a frequency of 1 Hz using field stimulation (32 mA, 40 ms, DS3 Isolated Current Stimulator, Digitimer Ltd., United Kingdom). The TCS SP-8 STED confocal microscope (Leica Microsystems) was used in the line scan mode (2.5 ms per line, 8000 lines, excitation 500 nm, emission 510–570 nm) to record fluo-3 fluorescence images in parallel with the transmission images. Thus, calcium transients and the corresponding myocyte shortenings could be assessed simultaneously (Figure 1). In a subset of experiments, rapid perfusion with Caffeine solution was applied after the series of electrical stimuli to evoke maximal calcium release and to assess the sarcoplasmic reticulum calcium content.

The transmission images were used to determine changes in sarcomere length using the SarConfoCal plugin (Pasqualin et al., 2017) in the Fiji environment (Schindelin et al., 2012) of ImageJ (Schneider et al., 2012). The adjacent averaging filter was set to 17. Only the intracellular regions of the images were analyzed.

The time courses of fluorescence and sarcomere length were normalized to the diastolic values (ΔF/F_0_, ΔL/L_0_), and imported into the Clampfit software (pClamp 10, Molecular Devices, United States). Here, the response to each stimulus, i.e., the calcium transient and its corresponding sarcomere length transient was analyzed. The following parameters were determined for calcium transient and sarcomere length transient: baseline, amplitude, half-width, rise time (20–80%), and decay time (80–20%). Smoothing window was set to five samples.

### Data Processing

Current–voltage data were fitted by the equation

(1) $$I_{Ca}\;(V)=g_{\max}(V-V_{r})/{\left(1+\exp(-(V-V_{1/2a})/V_{\mathrm{Sa}})\right)}^{4}$$

where *I_Ca_* is the peak calcium current amplitude, *V* is the test pulse voltage, g_max_ is the maximal conductance, V_r_ is the reversal potential, V_1/2a_ is the potential of half-activation, and V_Sa_ is the slope factor of activation.

Inactivation curves of the two-pulse inactivation protocols were fitted by the respective Boltzmann functions:

(2) $$I_{Ca}\;(V_{p})=-F_{\mathrm{iV}}/(1+\exp((V_{p}-V_{1/2i})/V_{\mathrm{SiV}}))+F_{\mathrm{iV}}$$

where *V_p_* is the prepulse voltage, F_iV_ is the inactivated fraction of the peak *I_Ca_* amplitude at the test pulse, V_1/2i_ is the half-inactivation potential, and V_SiV_ is the slope factor, and

(3) $$I_{Ca}\;(t_{p})=-F_{\mathrm{it}}/(1+\exp((t_{p}-t_{1/2i})/V_{\mathrm{Sit}}))+F_{\mathrm{it}}$$

where *t*_p_ is the prepulse duration, F_it_ is the inactivated fraction of the peak *I_Ca_* amplitude at the test pulse, t_1/2i_ is the prepulse duration for half-inactivation, and V_Sit_ is the slope factor.

Statistical analysis was performed in Origin (OriginLab, Ver. 9) using Student’s *t*-test. The *P*-values were calculated for two-tailed data distributions.

Data fitting was performed in Origin using the Levenberg–Marquardt method. The fitted parameters of two datasets were considered significantly different if their 95% confidence intervals did not overlap.

## Results

### Invalidation of Wolframin Does Not Lead to Heart Disease or Diabetes at the Age of 4 Months

The genotype of all experimental animals was verified using PCR. Overall examination of animals and their hearts excised for experiments did not show malformations or differences in comparison with standard rats. The weight of Wfs1^-e5/-e5^ rats taken into experiments was significantly smaller (524 ± 11 g, *n* = 19, *P* < 0.05) than that of their Wfs1^+/+^ littermates (570 ± 19 g, *n* = 17). Similar values and differences in the body weight at the age of 4 months were reported by Plaas et al. (2017). Tests for postprandial blood glucose showed slightly but significantly higher glycemia in Wfs1^-e5/-e5^ (7.79 ± 0.47 mmol/l, *n* = 8) than in Wfs1^+/+^ animals (6.10 ± 0.16, *n* = 6, *P* = 0.009) but no hyperglycemia in either group, in agreement with the original study for this age group (Plaas et al., 2017). This confirms that the cohort of experimental animals used in this study was not biased by specific or unspecific side effects related to breeding.

Echocardiographic examination did not reveal myocardial hypertrophy, infarction, or other major changes in the studied hearts. Quantitative evaluation provided similar parameters in both groups (*n* = 6 for Wfs1^+/+^ and *n* = 8 for Wfs1^-e5/-e5^, Table 1). The posterior wall thicknesses, either diastolic or systolic, were unchanged. Functional parameters showed some small differences between groups. The heart rate was somewhat higher, while the end-diastolic and stroke volumes were somewhat smaller in Wfs1^-e5/-e5^ than in Wfs1^+/+^ animals; these differences did not reach statistical significance. The aortic valve maximal flow velocity was significantly higher in Wfs1^-e5/-e5^ than in Wfs1^+/+^ animals, although its time integral was not different in the two groups. Based on measurements of the ejection fraction, which did not show significant differences between the two groups of animals, systolic function in Wfs1^-e5/-e5^ and Wfs1^+/+^ animals was fully preserved. At the organ level, therefore, the heart function of Wfs1^-e5/-e5^ animals can be considered compensated at this stage of disease development.

**Table 1:** Echocardiographic parameters.

	Wfs1^+/+^ (*n* = 6)	Wfs1^-e5/-e5^ (*n* = 8)	*P*
Heart rate (1/min)	307.4 ± 19.1	340.7 ± 15.9	0.20
PWd (mm)	1.74 ± 0.02	1.75 ± 0.08	0.87
PWs (mm)	2.43 ± 0.09	2.46 ± 0.11	0.84
EDV (cm^3^)	1.35 ± 0.09	1.16 ± 0.08	0.14
SV (cm^3^)	1.02 ± 0.06	0.90 ± 0.06	0.23
EF (%)	75.8 ± 1.6	77.4 ± 0.7	0.32
AV_max_ (mm/s)	1779 ± 85	2165 ± 141	0.05
AV VTI (mm)	92.4 ± 5.0	100.5 ± 6.7	0.38
MV E/A	1.30 ± 0.12	1.35 ± 0.10	0.74


*PWd – posterior wall thickness (diastolic), PWs – posterior wall thickness (systolic), EDV – end diastolic volume, SV – stroke volume, EF – ejection fraction, AV_*max*_ – aortic valve maximal flow velocity, AV VTI – aortic valve velocity time integral, MV E/A – mitral valve early (E)/late (A) peak velocity ratio, *n* – the number of animals, *P* – *P*-value of the *t*-test for two-tailed distribution.*

### Invalidation of Wolframin Leads to Elevated Contraction and Prolonged Calcium Transients

Contractile and calcium transient responses of isolated cardiac myocytes to field or caffeine stimulation (six Wfs1^+/+^ and eight Wfs1^-e5/-e5^ rats) were evaluated by optical methods of transmission and fluorescence microscopy, respectively (Figure 1). The resting sarcomere length was the same in both studied groups (Table 2); however, the contractile responses were different. The amplitude of peak contraction as well as the duration of contraction at the half-peak amplitude were significantly higher in Wfs1^-e5/-e5^ than in Wfs1^+/+^ myocytes. Since the rise time and the decay time of contraction did not change, prolongation of contraction resulted from prolonged duration of the plateau. Since contractions were recorded in the unloaded isotonic mode, they do not reflect metabolic or energy supply aspects of myocyte function.

**Table 2:** Parameters of cell shortening.

	Wfs1^+/+^ (*n* = 23)	Wfs1^-e5/-e5^ (*n* = 30)	*P*
Sarcomere length (μm)	1.72 ± 0.01	1.73 ± 0.01	0.23
Peak fractional contraction (ΔL/L_0_)	0.094 ± 0.006	0.112 ± 0.005	0.03
FDHM (ms)	224 ± 6	256 ± 7.7	0.004
Rise time 20–80% (ms)	75.1 ± 2.1	76.7 ± 1.7	0.54
Decay time 80–20% (ms)	101 ± 4	107 ± 3.5	0.26


*n – The number of analyzed cells. Data are given as mean ± SEM. *P* – *P*-value of the *t*-test for two-tailed distribution.*

In contrast to contractions, the respective peak amplitudes of stimulated calcium transients in Wfs1^-e5/-e5^ myocytes did not differ from those in Wfs1^+/+^ myocytes; however, their duration in Wfs1^-e5/-e5^ myocytes was significantly longer than in Wfs1^+/+^ myocytes (Table 3). The prolongation of contractions correlated well with prolongation of calcium transients, resulting in similar increases in FDHM (10 vs. 12%, respectively, Tables 2, 3).

**Table 3:** Parameters of calcium transients.

Field stimulation	Wfs1^+/+^ (*n* = 36)	Wfs1^-e5/-e5^ (*n* = 55)	*P*
Peak calcium transient (ΔF/F_0_)	3.02 ± 0.13	2.77 ± 0.13	0.20
FDHM (ms)	171 ± 3	188 ± 3	0.0001
Rise time 20–80% (ms)	24.9 ± 1.6	23.0 ± 1.3	0.34
Decay time 80–20% (ms)	129 ± 2	136 ± 3	0.09

**Caffeine**	**Wfs1^+/+^ (*n* = 7)**	**Wfs1^-e5/-e5^ (*n* = 14)**	

Peak calcium transient (ΔF/F_0_)	4.16 ± 0.26	4.29 ± 0.18	0.70


*n – The number of analyzed cells. Data are given as mean ± SEM. *P* – *P*-value of the *t*-test for two-tailed distribution.*

Stimulation of isolated myocytes by caffeine is a useful tool for estimation of calcium content of sarcoplasmic reticulum. Calcium transients in response to caffeine application were not different between groups (Table 3), indicating similar calcium content of the sarcoplasmic reticulum. Caffeine-induced contractures were always of supramaximal amplitude (unloaded cells) and therefore their kinetics were not evaluated.

### Invalidation of Wolframin Affects Release-Dependent Inactivation of Calcium Channels

**FIGURE 2:**
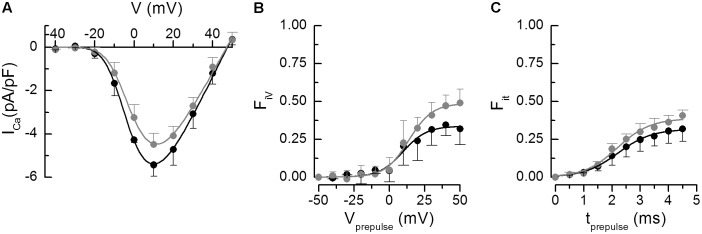
Analysis of calcium currents. The parameters of the fitted curves are in Table 4. **(A)** Voltage dependence of the peak calcium current amplitudes. The curves represent the respective fits by Eq. 1. **(B)** Fractional inactivation of the test pulse calcium current amplitude by variable prepulse voltages of 5-ms duration. The curves represent the respective fits by Eq. 2. **(C)** Fractional inactivation of the test pulse calcium current amplitude by prepulses to +60 mV of variable duration. The curves represent the respective fits by Eq. 3. In all panels, black – Wfs1^+/+^, gray – Wfs1^-e5/-e5^. Data are given as mean ± SEM (the number of cells *n* = 4, 4, and 2 for **A**, **B**, and **C**, respectively).

The change in contractility could result from a change of the calcium current (the trigger for calcium release) and/or from a change of calcium release itself. We estimated both calcium signals in isolated cardiac myocytes from 6 Wfs1^+/+^ and 8 Wfs1^-e5/-e5^ rats by means of a voltage-clamp experiment (Zahradníková et al., 2004). The voltage dependence of calcium currents is shown in Figure 2A together with the fitted curves according to Eq. 1. No statistically significant differences between parameters of the current–voltage curves of the two myocyte groups were observed. The parameters of the fit are summarized in Table 4.

**Table 4:** Parameters of calcium currents and the release dependent inactivation depicted in Figure 2.

Figure 2A	Wfs1^+/+^ (*n* = 40)	Wfs1^-e5/-e5^ (*n* = 40)
g_max_ (pA/pF/mV)	0.18 ± 0.02	0.17 ± 0.02
V_r_ (mV)	47.4 ± 1.7	47.0 ± 1.7
V_1/2a_ (mV)	-14.7 ± 1.8	-13.5 ± 2.0
V_Sa_ (mV)	8.9 ± 1.8	9.4 ± 2. 1

Figure 2B	**Wfs1^+/+^ (*n* = 44)**	**Wfs1^-e5/-e5^ (*n* = 44)**

F_iV_	0.34 ± 0.02	0.49 ± 0.02^∗^
V_1/2i_ (mV)	9.6 ± 2.1	13.7 ± 1.3
V_SiV_ (mV)	8.3 ± 1.7	8.3 ± 1.0

Figure 2C	**Wfs1^+/+^ (*n* = 20)**	**Wfs1^-e5^/^-e5^ (*n* = 20)**

F_it_	0.32 ± 0.01	0.39 ± 0.02^∗^
t_1/2i_ (ms)	2.20 ± 0.05	2.18 ± 0.10
V_Sit_ (ms)	0.60 ± 0.04	0.58 ± 0.08


*For Figure 2A: the best fit parameters using Eq. 1: g_*max*_ – conductance, V_*r*_ – reversal potential, V_*1/2a*_ – potential of half-activation, V_*Sa*_ – slope factor of the voltage-dependent activation*.

*For Figure 2B: the best fit parameters using Eq. 2: F_*iV*_ – maximum fraction of I_*Ca*_ inactivated by 5-ms prepulses, V_*1/2i*_ – prepulse potential for half-inactivation, V_*SiV*_ – slope factor of the inactivation curve.*

*For Figure 2C: the best fit parameters using Eq. 3: F_*it*_ – maximum fraction of I_*Ca*_ inactivated by +60-mV prepulses, t_*1/2i*_ – prepulse duration for half-inactivation, V_*Sit*_ – slope factor of the inactivation curve.*

*n – The number of fitted data points. ^∗^Significantly different from Wfs1^+/+^.*

The efficiency of calcium current to induce calcium release was analyzed according to Zahradníková et al. (2004). The extent of calcium release was estimated from the extent of calcium release-dependent inactivation of calcium current. The fraction of inactivated current (F_i_) was assessed by two two-pulse protocols. First, inactivation of I_Ca_ was assessed by Protocol 1, in which the potential of the 5-ms prepulse was varied. The dependence of F_iV_ on prepulse potential is shown in Figure 2B, and parameters of the curves fitted using Eq. 2 are given in Table 4. The voltage parameters of inactivation were not significantly different but the fraction F_iV_ of inactivated calcium current was significantly larger in Wfs1^-e5/-e5^ than in Wfs1^+/+^ myocytes. In the second two-pulse protocol experiment, calcium release was triggered by prepulses to +60 mV with variable duration that activated brief tail calcium currents of variable amplitude, known to induce only the calcium release-dependent component of inactivation (Zahradníková et al., 2004). The subsequent test pulse to 0 mV was used to assess the amplitude of calcium current that was not inactivated by calcium release (Protocol 2). The dependence of the fraction of inactivated current, F_it_, on the prepulse duration is shown in Figure 2C and the parameters of the curves fitted using Eq. 3 are shown in Table 4. The parameters of inactivation were not significantly different between the two myocyte groups except for the fraction F_it_, which was again significantly elevated in Wfs1^-e5/-e5^ myocytes.

These data show that the fraction of calcium current inactivated by short prepulses is increased in Wfs1^-e5/-e5^ animals, despite no observed changes in the amplitude or the voltage-dependence of the calcium current itself. Since neither the half-inactivation nor slope factors of prepulse-induced I_Ca_ inactivation were changed, these data suggest that the voltage dependence of I_Ca_ inactivation was not affected by wolframin invalidation. To summarize, these two-pulse experiments revealed an increased effect of calcium release on calcium current inactivation in Wfs1^-e5/-e5^ myocytes relative to Wfs1^+/+^ myocytes. This could be caused only by increased calcium release at individual dyads.

### Invalidation of Wolframin Does Not Affect Ultrastructure of Cardiomyocytes

**FIGURE 3:**
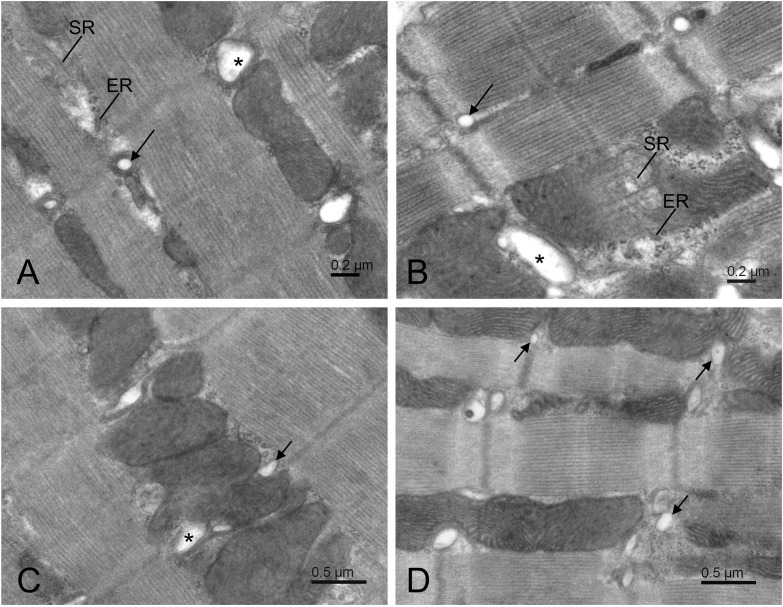
Electron micrographs of typical central area of Wfs1^+/+^ **(A,C)** and Wfs1^-e5/-e5^ **(B,D)** cardiomyocytes. Long arrows and asterisks indicate dyads containing small and large t-tubule profiles, respectively. Short arrows indicate non-dyadic t-tubules, that is, those without apposed terminal cisternae. ER – rough and SR – smooth reticular membranes.

Electron microscopic examination of ultrastructure of cardiomyocytes (five Wfs1^+/+^ and five Wfs1^-e5/-e5^ rats) revealed very similar morphology in both groups of experimental animals (Figure 3). The myocytes displayed standard shape, contractile myofibrils were well organized, and mitochondria formed columns between myofibrils. The ribosomes were present in the cytosol between mitochondria and myofibrils. Both the rough and smooth reticular membrane systems occurred in the intermyofibrillar space. Cardiomyocytes of both groups contained dyads near Z-lines. According to visual examination, the diameter of t-tubule profiles in dyads seemed somewhat different between groups, albeit not substantially (Figure 3A,B). The two groups seem to differ by higher occurrence of small non-dyadic t-tubules near Z-lines in the Wfs1^-e5/-e5^ rats in comparison to Wfs1^+/+^ (Figure 3C,D).

### Invalidation of Wolframin Does Not Affect Protein Expression of SERCA

**FIGURE 4:**
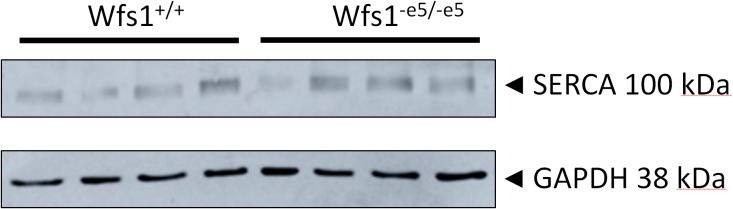
Expression of SERCA2 protein in cardiac myocytes of Wfs1^+/+^ (*n* = 4) and Wfs1^-e5/-e5^ (*n* = 4) animals. GAPDH was used as a loading control.

To determine whether invalidation of wolframin affects expression of SERCA, its protein levels in myocytes (six Wfs1^+/+^ and eight Wfs1^-e5/-e5^ rats) were determined by western blots using the housekeeper protein GAPDH as a loading control (Figure 4). The analysis yielded values of 0.44 ± 0.07 vs. 0.45 ± 0.06 a.u. for Wfs1^+/+^ and Wfs1^-e5/-e5^, respectively (mean ± SEM, *n* = 4), indicating no difference between protein expression of SERCA of either group.

## Discussion

In this work, we studied the effect of invalidation of wolframin on cardiac function using exon5-Wfs1 deficient rats (Wfs1^-e5/-e5^), with emphasis on calcium signaling related to contractility of cardiac myocytes. At the level of whole heart, we used echocardiography to assess cardiac functional parameters. At the level of isolated ventricular myocytes, we characterized cell contraction and intracellular calcium transients together with calcium currents. In addition to genotyping and blood glucose level verification, we examined changes in the expression of SERCA2 and in the ultrastructure of the left ventricular myocytes.

This is to our knowledge the first study of the effect of wolframin invalidation on calcium handling in cardiac myocytes. Previous works on wolframin were performed mostly in pancreatic β-cells and neurons or in cell lines. In those studies, the changes in cell function induced by wolframin invalidation or silencing went hand-in-hand with induction of ER stress (Takei et al., 2006; Hara et al., 2014; Zatyka et al., 2015; Cagalinec et al., 2016; Morikawa et al., 2017), with suppression of SERCA activity (Takei et al., 2006; Hara et al., 2014; Morikawa et al., 2017) and mRNA expression (Morikawa et al., 2017). Paradoxically, increase of SERCA protein expression was also observed (Zatyka et al., 2015). It has been proposed that the role of wolframin in pancreatic β-cells is to target SERCA to proteasome-mediated degradation (Zatyka et al., 2015). On the other hand, induction of ER stress by wolframin deficiency was not observed in either cardiac or skeletal muscle cells under conditions, when ER stress was clearly present in pancreatic β-cells (Yamada et al., 2006). It should be noted that in β-cells the role of SERCA is to damp the amplitude of cytosolic calcium oscillations during cell depolarization by sequestration to the ER (Félix-Martínez and Godínez-Fernández, 2014). In cardiac myocytes, SERCA primes calcium stores for stimulated calcium release and it returns calcium back to the store after the stimulus. It is therefore plausible that the role of wolframin in cardiac myocytes differs from that in pancreatic β-cells and neurons.

Ultrasonographic examination showed that the function of Wfs1^-e5/-e5^ hearts was fully compensated at this age of WS development, despite the lower body weight of Wfs1^-e5/-e5^ rats relative to control littermates. Additionally, no overt difference in the ultrastructure of ventricular myocytes or SERCA2 expression was observed between the two groups in our study. These findings comply with previous findings that symptoms of WS develop in Wfs1^-e5/-e5^ rats at the age of 13 months (Plaas et al., 2017). However, we have observed several differences in the function of cardiomyocytes between the two groups. Invalidation of wolframin led to increased release of calcium measured either as fractional inactivation of calcium current, or as prolongation of cytosolic calcium transients and myocyte contraction, or as increased amplitude of cell contraction in response to field stimulation. Interestingly, the amplitudes of calcium transients induced by caffeine were not different, suggesting that the SR content was similar in both myocyte groups. Together with no difference in expression of SERCA2, this indicates no effect of wolframin invalidation on calcium accumulation in the SR of Wfs1^-e5/-e5^ myocytes at the studied age of rats.

Prolonged cytosolic calcium transients at the same maximal intensity and prolonged contraction observed in Wfs1^-e5/-e5^ myocytes can be explained by the local control mechanism of calcium release (Cannell et al., 1994; Zahradníková et al., 2010; Janíček et al., 2012). According to this, the amplitude and the rate of cytosolic calcium increase result from temporal summation of individual release events arising at dyads and the number of dyads recruited from their cellular pool. The resulting contraction convolves with a certain delay the cytosolic calcium increase, the supply of ATP to drive the myosin motors, and sequestration of calcium to SR by SERCA.

It could be that in the field-stimulated isolated myocytes the observed differences occurred in part due to longer action potential duration in Wfs1^-e5/-e5^ myocytes. In this respect, patch-clamp experiments showed similar voltage dependent characteristics of calcium currents in both groups, i.e., no change in function of L-type calcium channels that could cause action potential prolongation. To the contrary, we observed increased calcium current inactivation due to calcium induced calcium release mechanism (Sham, 1997; Zahradníková et al., 2004) that should contribute to faster termination of the action potential. However, we could not exclude changes in potassium currents controlling termination of action potentials.

Our results show that calcium release in Wfs1^-e5/-e5^ is prolonged relative to that of Wfs1^+/+^ myocytes under conditions of identical activating stimuli. We speculate that the longer duration but unchanged intensity of calcium release might arise by a yet unknown mechanism from the regulatory effect of wolframin on RyR2 channel open time and/or termination of calcium release, or on modulation of the calcium signal by mitochondria. All of these pathways are plausible, since wolframin was shown to interact with calmodulin (Yurimoto et al., 2009), a regulator of RyR2 activity with a potential role in RyR refractoriness (Liu et al., 2018), and since wolframin deficiency affected mitochondrial function in cortical neurons (Cagalinec et al., 2016).

## Conclusion

We have shown that invalidation of Wfs1 results in subtle changes in calcium signaling, present before overt onset of disease. These subtle changes result in significant augmentation of both amplitude and duration of contraction. However, the sensitivity of our methods did not allow us to find the molecular basis of the observed changes. To resolve this question, further studies are necessary, such as direct measurements of calcium release flux from individual dyads, and/or analysis of expression and function of all proteins involved in calcium cycling. It is tempting to speculate on wolframin regulation of RyR2 gating, e.g., by prolongation of channel openings or by delaying calcium release termination. Moreover, since the structure, density, size, and distribution of dyads was recognized as an important player in proper function of cardiac calcium release, the role of wolframin in their formation is worth further study.

## Data Availability

The datasets generated for this study are available on request to the corresponding author.

## Author Contributions

MC, AZ, and IZ conceived of or designed the study. MC and AZ contributed to measurements of calcium transients and myocyte shortening. AZjr and MH contributed to electrophysiology. MN and IZ contributed to electron microscopy. DK and LP contributed to echocardiography. SK and AZjr contributed to western blotting. MC, JP, AZ, AZjr, and IZ analyzed the data. AZ, IZ, and MC wrote the manuscript. MP provided materials. MC, AZ, AZjr, and IZ edited the manuscript for important intellectual content.

## Conflict of Interest Statement

The authors declare that the research was conducted in the absence of any commercial or financial relationships that could be construed as a potential conflict of interest.
